# Supplementary material for: Chromothripsis during telomere crisis is independent of NHEJ, and consistent with a replicative origin
Source: Genome Res. 2019 May;29(5):737–49. doi: 10.1101/gr.240705.118 (PMC6499312; doi:10.1101/gr.240705.118)
Supplement: Supplemental Material [file supp_gr.240705.118_Supplemental_file_1.zip › contigs/annotated_contigs/DB75/contig.2.DB75_length_486_mean_cov_7.40740740741.docx]

**DB75_length_486_mean_cov_7.40740740741**

```
CATTCCAGATCTCAGATGTTTTCAGGACAACCCTCTCACCTAGTGAAACAGATTGTATGTTTTTTCCACTGTGGCTTGCTCCTATTTAT
 >chr4:33836107-33836237 - E=2e-50
TTCTATTTCCTTCAGCCCAGAAAGGATTATTGTAATAT|TGATATATTGATGTATTATTGATGTATAATTATGATATATTGATGTAATA

TTGATATATTA|CATCAGCATTTTTTAAATTCTTACATTAATCTATTTAAATTGAGGTCCTATTTTACCTCAAAGATATCTATTACCTG
            >chr7:57606809-57607113 - E=7e-130
TATTTATCTATCCAATCGAGTTCGTGCCACATATATTTTTTTTAATTCTGCAATTCCTCTGAAGTTTTTAAGATCTTTTCTTGCTGGTT

AGAATAATCCTCAAGTTTTTCTAATCATAGCACTTTTATTTTCTCATTCTTCCGATTGAAATTCTATCCTCTTCATTTACAAAGGTGAA

TATATTTGGAACTGTCATCTCCAATGAAAAGCAATTAGAAACC
```
